# Menstrual Cycle Phase Influences Cognitive Performance in Women and Modulates Sex Differences: A Combined Longitudinal and Cross-Sectional Study

**DOI:** 10.3390/biology14081060

**Published:** 2025-08-15

**Authors:** Angelika K. Sawicka, Katarzyna M. Michalak, Barbara Naparło, Adrià Bermudo-Gallaguet, Maria Mataró, Pawel J. Winklewski, Anna B. Marcinkowska

**Affiliations:** 1 Applied Cognitive Neuroscience Lab, Department of Neurophysiology, Neuropsychology and Neuroinformatics, Medical University of Gdansk, 80-211 Gdansk, Poland; rabarbara@gumed.edu.pl (B.N.); anna.marcinkowska@gumed.edu.pl (A.B.M.); 2 Departament de Psicologia Clínica i Psicobiologia, Facultat de Psicologia, Universitat de Barcelona (UB), Passeig de la Vall d’Hebron, 171, 08035 Barcelona, Spain; abermudo@ub.edu (A.B.-G.); mmataro@ub.edu (M.M.); 3 Institut de Neurociències, Universitat de Barcelona, Passeig de la Vall d’Hebron, 171, 08035 Barcelona, Spain; 4 Institut de Recerca Sant Joan de Déu, Santa Rosa 39-57, 08950 Esplugues de Llobregat, Spain; 5 Department of Neurophysiology, Neuropsychology and Neuroinformatics, Medical University of Gdansk, 80-211 Gdansk, Poland; pawel.winklewski@gumed.edu.pl; 6 Institute of Health Sciences, Pomeranian University in Slupsk, 76-200 Slupsk, Poland; 7 2nd Department of Radiology, Medical University of Gdansk, 80-211 Gdansk, Poland

**Keywords:** menstrual cycle, gonadal steroid hormones, memory short-term, attention, sex characteristics, oestradiol, follicular phase

## Abstract

Sex hormones’ and menstrual cycle’s effects on cognitive performance remain unclear. This study examined cognitive differences between women across menstrual cycle phases, sex differences between women and men, and hormone–cognition associations. In total, 71 healthy young adults, aged 20–36 (42 women, 29 men), completed standardised cognitive tests measuring attention, processing speed, working memory, and visuospatial abilities. Women were tested twice: during menstrual (low-oestradiol) and pre-ovulatory (high-oestradiol) phases; men once. Hormone levels (oestradiol, progesterone, testosterone) were measured in blood samples via electrochemiluminescence immunoassay (ECLIA). Two analytical strategies were used: (1) within-subject analysis comparing women between phases, and (2) between-group comparison across three groups—women in menstrual phase, pre-ovulatory phase, and men. Women performed better during pre-ovulatory versus menstrual phase in working memory (Digit span forward: *p* = 0.04; Digit span backwards max: *p* = 0.02) and attention switching (Trail Making Test B: *p* = 0.01). Sex differences in processing speed were observed only during the menstrual phase (Trail Making Test A: *p* = 0.03; Stroop B: *p* = 0.04), but not in the pre-ovulatory phase. Positive correlations between oestradiol/progesterone and cognitive performance were found in men, while complex bidirectional relationships emerged in women during the menstrual phase only. Testosterone showed no significant correlations. These findings highlight hormonal status effects on cognitive sex differences.

## Simple Summary

Hormonal fluctuations during the menstrual cycle can impact a woman’s performance on tasks that require memory, attention, and cognitive processing speed. These changes could also help explain cognitive differences between women and men. In this study, we tested 71 young adults on a series of cognitive tasks. Women were assessed twice: during the menstrual phase (low hormone levels) and the pre-ovulatory phase (when oestradiol is high). Men were tested once. We found that women performed better on memory and attention tasks just before ovulation. Differences in processing speed between men and women were observed only during women’s menstrual phase. These differences disappeared when oestradiol levels were higher (before ovulation). In men, better performance was also linked to higher oestradiol and progesterone. Our results suggest that oestradiol plays an important role in cognitive changes during the menstrual cycle. Recognising hormonal variation, especially in oestradiol, may be essential when studying sex differences in cognition.

## 1. Introduction

The menstrual cycle, characterised by dramatic fluctuations in sex hormone levels, provides a natural model for studying hormonal influence on cognition. The menstrual cycle begins with the early follicular phase, characterised by low progesterone and oestrogen levels. Oestrogen levels rise rapidly in the late follicular phase, showing a nearly eight-fold increase and a peak one day before ovulation. The luteal phase sees a steady rise in progesterone levels that peaks in the mid-luteal phase with an 80-fold increase, accompanied by a second oestrogen peak. Both hormone levels decline during the late luteal phase, reaching baseline shortly before the onset of menstruation [1,2].

Research shows that steroid hormones, their fluctuations, and their receptors play a significant role in various brain functions, including regulating socio-sexual behaviour, neurogenesis, cognitive function, mood, and emotion [3,4]. oestrogen receptors (ERs) and progesterone receptors (PRs) are found throughout the brain in regions involved in cognitive and emotional regulation [3,4,5]. Through these receptors, sex hormones influence cognitive function via multiple mechanisms, including the modulation of neurotransmitter systems, the regulation of synaptic plasticity, and effects on neural connectivity [6,7,8].

In young women, evidence suggests that hormonal fluctuations can induce reversible structural changes in the brain. Research has demonstrated that grey matter volume in young women exhibits relative increases in the right anterior hippocampus and relative decreases in the right dorsal basal ganglia during the postmenstrual phase [9]. It has been proven that oestrogen improves performance in prefrontal cortex-dependent learning in animal and human studies [10,11,12]. Related changes were seen in studies of healthy women showing differential Stroop task performance between phases characterised by low versus high concentrations of oestradiol and progesterone during the menstrual cycle [13]. These results suggest that sex-related hormone modulation selectively affects cognitive function depending on the type of task and that low levels of oestradiol secretion appear to contribute to a reduction in the level of attention related to the aforementioned prefrontal cortex. According to research, higher oestrogen levels have a protective effect on cognitive functioning [5] and positively correlate with processing speed and sustained attention [14]. Furthermore, oestrogen is involved in memory processes and can also affect different types of memory, such as episodic memory, working memory [15], and long-term memory [16]. Additionally, oestradiol may modulate visuospatial functions, including visuospatial orientation [17] and visuospatial memory [18]. Regarding progesterone, an fMRI study proved that this hormone modulates limbic and somatomotor networks, which can improve cognitive function in naturally cycling young women [19]. Both oestrogen and progesterone treatments have shown potential cognitive benefits in women, with progesterone showing better effects on verbal working memory [20]. Testosterone, on the other hand, activates a distributed cortical network, the ventral processing stream, during spatial cognition tasks, and the addition of testosterone improves spatial cognition in men [21]. According to research, testosterone also protects the brain against oxidative stress, serum deprivation-induced apoptosis, and soluble amyloid-β (Aβ) toxicity [22]. Recent research has suggested that testosterone’s effects on cognition may be particularly important in women, especially in those carrying genetic risk factors for cognitive decline [23]. This hormone can be converted to oestradiol in the brain through aromatisation, thereby potentially affecting cognition through both androgen and oestrogen-dependent mechanisms [24]. Studies have suggested the involvement of testosterone in spatial abilities and working memory, though its effects may differ between men and women [24,25].

However, despite numerous studies suggesting that cognitive functions in women vary depending on the phase of the menstrual cycle and hormone levels, the scientific literature remains inconsistent, and several studies have not found significant changes [26,27,28]. These contradictory findings may stem from methodological differences, including reliance on estimated cycle phases rather than quantitative hormonal measurements, variations in cognitive testing batteries employed, and differences in participant characteristics and sample sizes.

In summary, steroid hormones, especially oestradiol, may influence cognitive function in areas critical for daily functioning and academic achievement, making them particularly relevant for the study of hormone-dependent changes in cognitive function in young adult women. However, the exact nature of these relationships within the menstrual cycle remains unclear, especially regarding their potential impact on sex differences in cognitive functioning between women and men.

Building on the existing literature, we aimed to investigate three key research questions. First, we examined whether women’s cognitive functioning changes across the menstrual cycle phases, focusing on attention, processing speed, short-term and working memory, and visuospatial abilities. We hypothesised that performance would be enhanced during the pre-ovulatory phase (high oestradiol) compared to the menstrual phase (low oestradiol and progesterone), reflecting primarily the facilitating effects of elevated oestradiol levels, particularly in tasks measuring working memory [10,12], processing speed [14], and attention [19]. Second, we investigated sex differences in cognitive functioning between young, healthy women and men, comparing men with women in both menstrual cycle phases to determine whether these differences are modulated by hormonal status. Here, we expected to find sex differences in information processing speed and visuospatial abilities, with these differences being dependent on women’s menstrual cycle phase [17,21]. Third, we explored associations between sex hormone levels (oestradiol, progesterone, and testosterone) and cognitive performance in both men and women, comparing the menstrual phase (lowest oestradiol) with the pre-ovulatory phase (highest oestradiol). For this aim, we hypothesised that higher oestradiol levels would be associated with better working memory and attention performance [10,12], progesterone levels would show an association with cognitive performance, in line with previous research, through underlying neural mechanisms (the limbic and somatomotor networks) [19], and testosterone levels would demonstrate positive relationships with spatial abilities and working memory [24,25].

In this study, we specifically chose to compare the menstrual phase (days 2–5 after menstruation onset) and the pre-ovulatory phase (up to 2 days before expected ovulation) for several methodological and theoretical reasons. These two phases represent the most distinct hormonal profiles within the menstrual cycle, with the menstrual phase characterised by minimal levels of both oestradiol and progesterone, while the pre-ovulatory phase features a pronounced oestradiol peak with still relatively low progesterone. This hormonal contrast provides an optimal window to examine oestradiol’s specific effects on cognition with minimal confounding influence from progesterone.

To address these research questions, the study employed two analytical approaches: (1) a longitudinal analysis including only women, comparing their cognitive performance across the menstrual and pre-ovulatory phases, and (2) a cross-sectional analysis comparing men and women at each phase. By combining these two approaches, we provided new insights into sex differences and hormonal fluctuations within the same examination group. By measuring hormone levels, we could exclude nonspecific patients with irregular cycles or without hormone peaks fitting the normal range for the cycle phase. For methodological consistency in the latter approach, only data from women’s first evaluation sessions were used compared with men.

## 2. Materials and Methods

### 2.1. Ethics Statement

All participants were informed about the procedures, risks, and expected outcomes before starting the assessment procedure and gave their written informed consent for participation. The study was conducted under the Declaration of Helsinki. The study protocol was approved by the Independent Bioethics Commission for Research at the Medical University of Gdansk (NKBBN/398/2021 and NKBBN/398-14/2023).

### 2.2. Participants

**Figure 1:**
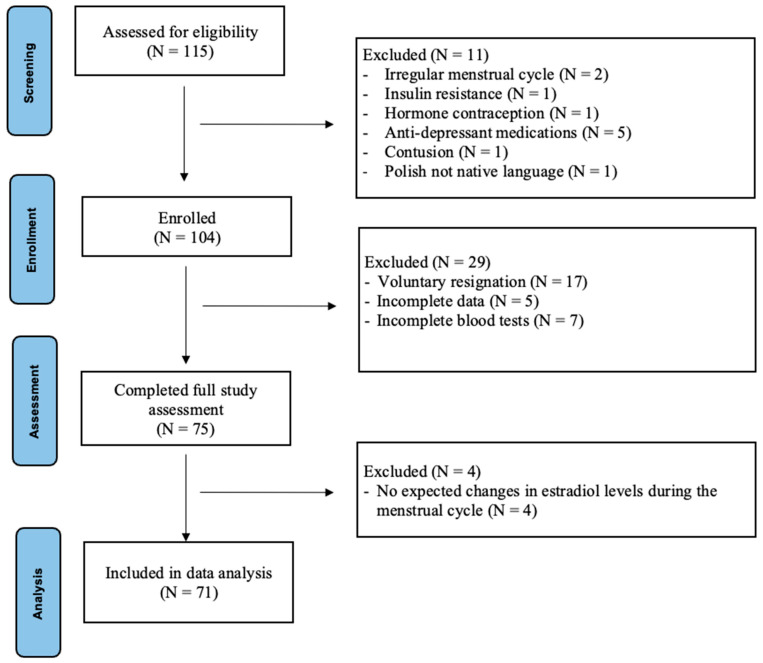
Trial flow diagram. Note: The diagram illustrates the recruitment, screening, and selection process for all participants in the study. The final sample, consisting of 71 participants, comprised 42 women and 29 men.

Recruitment for the study was conducted continuously between December 2022 and November 2023. The participants were recruited through the universities’ methods of communication, such as mailing lists and advertisements on social media. The qualifications for the study were assessed using an online questionnaire and consultation. A diverse group of 115 people applied for the study, of whom 104 were accepted for the study procedure. Inclusion criteria for all participants were as follows: an age range of 18–36 years and being a native Polish speaker. For women, an additional inclusion criterion was a regular menstrual cycle length defined as 24 to 38 days (as defined by the International Federation of Gynaecology and Obstetrics (FIGO) in 2018), with a variation in duration between cycles of no more than 8 days [29]. The non-inclusion criteria were as follows: any neurological or mental disorder, current use of psychiatric medications, chronic diseases (such as diabetes), irregular menstruation in women, endometriosis or polycystic ovary syndrome, current or recent (within the past six months) use of hormonal contraceptives, current or past hormone therapy, current pregnancy, and postpartum period or breastfeeding within one year prior to the study. In addition, women who showed inconsistency between their declared cycle phase and measured hormone levels were excluded from the statistical analysis. Ultimately, 71 young, healthy adults were included in the statistical analyses—42 women (mean age = 23.64 ± 3.53) and 29 men (mean age = 24.1 ± 3.46). All participants had comparable education levels (mean years of education = 16.02 ± 2.48) and body mass index (BMI; mean = 23.09 ± 3.72), ensuring a high degree of group homogeneity (Figure 1).

### 2.3. Study Design

**Figure 2:**
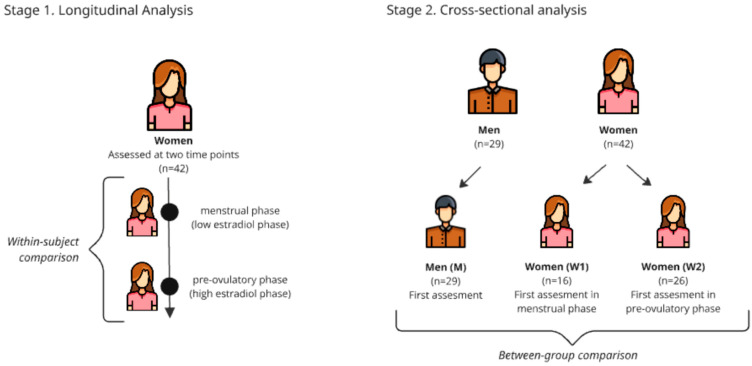
Study design overview: Two-stage analytical approach. Note: Stage 1—Longitudinal Analysis (within-subject comparison): Women (n = 42) were assessed at two time points within the same menstrual cycle (during the menstrual phase (low oestradiol) and pre-ovulatory phase (high oestradiol)), allowing for within-subject comparisons of cognitive performance across hormonal states. Stage 2—Cross-sectional Analysis (between subject comparison): For sex difference analyses, participants were reorganised into three independent groups based on the timing of the first cognitive assessment: Men (M; n = 29) who underwent a single assessment, Women (W1; n = 16) whose first assessment occurred during the menstrual phase, and Women (W2; n = 26) whose first assessment occurred during the pre-ovulatory phase. This approach enabled between-group comparisons while controlling for practice effects by using only first-session data from women when comparing with men.

To address our research questions, two main analytical approaches were used (Figure 2).

(1) Longitudinal analysis (within women): To compare women’s cognitive functioning between the menstrual and pre-ovulatory phases, the Wilcoxon signed-rank test with a calculated effect size was used for dependent groups.

(2) Cross-sectional analysis (between men and women): To compare cognitive performance between men and women, participants were divided into three independent groups: men (M; n = 29), women in the menstrual phase (W1; n = 26), and women in the pre-ovulatory phase (W2; n = 16). The groups of women (W1 and W2) were established based on the phase in which they underwent their first neuropsychological assessment to ensure methodological homogeneity and avoid practice effects when comparing with men, who were tested only once. The Kruskal–Wallis test, followed by post hoc Mann–Whitney U tests, was used to evaluate differences between these three groups.

### 2.4. Assessment Timing

To precisely determine assessment periods coinciding with specific hormonal states, we implemented a structured menstrual cycle tracking protocol. Female participants completed a reproductive history questionnaire documenting cycle regularity, average cycle length (calculated from the preceding three menstrual cycles), and the onset date of their most recent menstruation. All women included in the final analysis exhibited regular menstrual cycles (ranging between 24 and 38 days) [29].

Assessments were scheduled during two distinct cycle phases characterised by maximally differentiated oestradiol profiles: the menstrual phase (days 2–5 post-menstruation onset), corresponding to minimal oestradiol and progesterone concentrations; and the pre-ovulatory phase (0–2 days pre-ovulation), characterised by elevated oestradiol with still relatively low progesterone levels [30]. Expected ovulation dates were calculated using the reverse counting method (subtracting 14 days from the anticipated next menstruation) and verified through confirmation of subsequent menstrual onset.

To control for potential practice effects while ensuring within-subject comparisons, we employed a randomised crossover design in which all participants completed two assessments—one during the menstrual phase and one during the pre-ovulatory phase. Participants were randomly assigned to begin the study in either cycle phase, with their second assessment scheduled in the alternate phase. This approach enabled us to follow each woman across both hormonal conditions while controlling for potential order effects. Following participant attrition and exclusion of cases with hormonal profiles inconsistent with expected phase values, the final analytical sample included 42 women (26 who began in the menstrual phase and 16 who began in the pre-ovulatory phase). This design supports robust within-subject comparisons while mitigating confounding practice effects.

### 2.5. Hormone Measurements

Before every cognitive examination, blood samples were collected from the participants to determine their hormone levels of progesterone, oestradiol, and testosterone. For female participants, blood samples and cognitive tests were conducted twice, timed to capture hormonal fluctuations across the menstrual cycle (once during the menstrual phase and once during the pre-ovulatory phase). For male participants, blood sampling and cognitive testing were performed once.

Blood tests were performed in the fasting state and collected in the morning, specifically between 7:00 and 10:00 a.m. The samples were collected and analysed by a certified commercial laboratory using the electrochemiluminescence immunoassay (ECLIA) method and Cobas Pro device.

The analytical performance characteristics for the assays were as follows: for oestradiol, the limit of detection (LoD) was 18.4 pmol/L (5 pg/mL), the limit of quantification (LoQ) was 91.8 pmol/L (25 pg/mL), intra-assay CV ranged from 1.2 to 5.4%, and inter-assay CV ranged from 1.9 to 7.1%. For progesterone, the LoD was 0.159 nmol/L (0.05 ng/mL), the LoQ was 0.636 nmol/L (0.2 ng/mL), intra-assay CV ranged from 1.3 to 3.2%, and inter-assay CV ranged from 3.7 to 5.5%. For testosterone, the LoD was 0.087 nmol/L (0.025 ng/mL), the LoQ was 0.416 nmol/L (0.120 ng/mL), intra-assay CV ranged from 1.1 to 3.0%, and inter-assay CV ranged from 2.3 to 5.1%. Precision was determined according to CLSI (Clinical and Laboratory Standards Institute) protocol EP05-A3.

### 2.6. Cognitive Tests

Cognitive testing was conducted between 8:00 a.m. and 12:00 p.m. to ensure consistency in timing and to control for potential circadian influences. During the neuropsychological assessment, the participants completed six tests in a fixed order, as described below. The total duration of the cognitive assessment was 45–60 min, depending on each individual’s performance speed. Short breaks (2–3 min) were provided between the tests when requested by the participants to minimise fatigue effects.

The Stroop test from the Delis–Kaplan Executive Function System (D-KEFS) battery [31] measures rapid processing, attentional selectivity, inhibitory processing, and cognitive flexibility. It is a neuropsychological test widely used to assess the ability to inhibit cognitive interference, which occurs when the processing of one stimulus feature prevents the simultaneous processing of a second stimulus feature. This test contained four conditions, each preceded by a short trial, and the time taken to complete each was measured. The first task (A) was to say the colour of the squares (blue, red, and green), the second (B) was to read words written in black ink as quickly as possible, the next (C) was to say the colour of the ink in which the words were written (written colour names were incongruent with the ink colour), and the final task (D) was to read a word written in an ink colour incongruent with the name of the colour if written without a frame, or to say the name of the colour according to the word that was written if the word was in a frame. All the tasks were presented on white sheets of A4 paper lying horizontally. Word reading and colour naming are measures of processing speed, while colour–word inhibition measures executive functions [32]. In this test, we measured the time taken to complete each task and the interference between each subtest.

Digit span forward and backwards repetition from the Wechsler Adult Intelligence Scale [33] measures auditory short-term and working memory. The participants repeated an increasing number of random digits forward and then backwards in the order given by the researcher. Each correctly repeated series was followed by another series plus an additional digit. If the participant failed the first attempt, the subject was given a second chance with a different set of numbers of the same length. If the subject failed the second attempt, the test was terminated. In this test, we measured the number of points the subject scored and the number of items correctly recalled in the longest sequence.

The trail making test (TMT) parts A and B [34] measure visual processing speed, visual perceptual ability, working memory, task-switching ability, and executive control. In part A, the participants had to match the following numbers on an A4 sheet of paper; in part B, the numbers and letters alternated in alphabetical order. Before the actual test, the participants completed a trial task. The time taken to complete the task was measured. Another indicator of working memory performance was the ratio of the time taken to complete part A to part B.

The Corsi block-tapping test [35] measures spatial short-term and working memory. The test requires the maintenance of a visuospatial pattern and sequence of movements [36]. Corsi’s original apparatus consisted of a series of nine blocks arranged irregularly on a board. The blocks were tapped by the experimenter in random sequences of increasing length. There were two subtests: the forward and the backwards subtest. Immediately after each tapped sequence, the participant attempted to reproduce it, progressing until they failed to correctly reproduce two sequences of the same length [37]. As the test progressed, the number of blocks increased. The score was assessed by the maximum number the participant could reproduce correctly in the forward and backwards directions. The total score forward (TSF) and total score backwards (TSB) indices gave the number of examples performed correctly multiplied by the length of the sequence reproduced correctly.

The visual pattern test (VPT) [38] measures short-term non-verbal memory and memory for item sequences. There were two parallel sets of patterns, set A and set B, which formed two parallel forms of the test, version A and version B, respectively. The grids ranged in size from the smallest, a 2 × 2 matrix (with two filled cells), to the largest, a 5 × 6 matrix (with 15 filled cells), with the complexity increasing progressively by adding two additional cells to the previous grid. Therefore (assuming the simplest pattern can be reproduced), the subject received a score ranging from 2 to 15 [39]. During the test, the participants were shown patterns of black squares for three seconds and asked to reproduce them from memory. The test was stopped when the participant incorrectly reproduced three boards with the same number of cells. The result of the test was the maximum number of elements that the participant was able to recall and the average number of the last three examples that the participant got right.

The visual perceptual skills–subtests memory and sequence [40] measure short-term visual recognition. Due to the young, healthy group of participants, the first six items in each subtest were omitted—the items were too easy to recognise and did not differentiate. The next 10 items were shown for three seconds, and the last six for 5 s. After viewing each figure or sequence of elements, the participant was expected to identify and select the correct one from other similar options. A maximum of 18 points could be scored. During the test, the participant did not receive any feedback on whether they were speaking correctly.

### 2.7. Statistical Analysis

Statistical analyses were performed using the Statistical Package for Social Sciences (SPSS) version 29 (IBM Corp., Armonk, NY, USA) and GraphPad Prism 8 (GraphPad Software, San Diego, CA, USA).First, Shapiro–Wilk’s test was used to check for the normal distribution of the variables. As the cognitive results and hormonal concentrations were not *normally* distributed, non-parametric tests were performed.

For the longitudinal part of the study, women’s cognitive performance in the two phases of the menstrual cycle was compared using the Wilcoxon signed-rank test with a calculated effect size. For the cross-sectional part of the study, women’s cognitive performance in the two phases of the menstrual cycle was compared to men’s using the Kruskal–Wallis test with a post hoc Mann–Whitney U test.

Correlations between the changes in hormone levels and cognitive performance were calculated using partial correlation tests adjusted by age. To assess the relationship between hormone levels and cognitive performance, we matched each participant’s hormone concentrations to their performance on specific cognitive tasks completed on the same day. Partial correlations were conducted for all samples together and separately for each group (M, W1, and W2). A *p*-value < 0.05 was considered statistically significant.

## 3. Results

### 3.1. Hormonal Data

**Figure 3:**
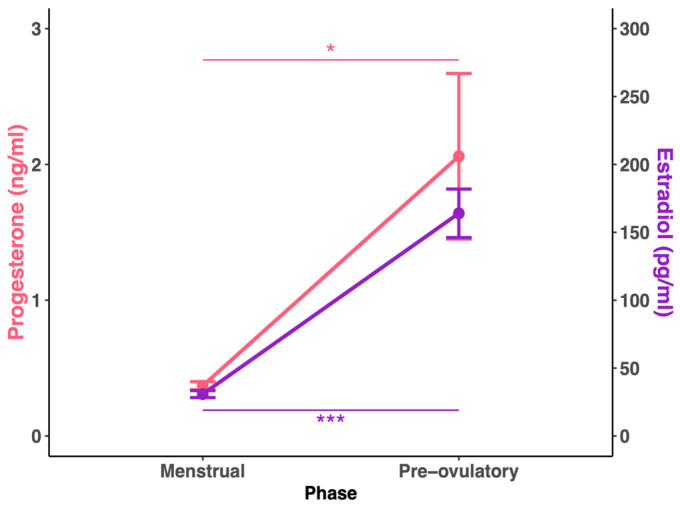
Changes in progesterone and oestradiol levels in women (n = 42) during two menstrual cycle phases. *Note:* Data are presented as the mean ± SE and were analysed using the Wilcoxon signed-rank test. Significant differences are denoted by *** *p* < 0.001 and * *p* < 0.05. Progesterone is marked in coral, while oestradiol is marked in violet.

Women participating in the study were characterised by a regular menstrual cycle, with a duration in our group ranging from 25 to 35 days (Table 1). Oestradiol levels were significantly higher in the pre-ovulatory phase than in the menstrual phase of the cycle (T = 5.65; *p* < 0.001; r = 0.6), and progesterone levels were likewise higher (T = 2.48; *p* = 0.01; r = 0.3) (Figure 3). Hormonal status was as expected for the healthy women in both phases of the menstrual cycle (Table 2). We also investigated hormone levels in men during the study (Table 2).

### 3.2. Changes in Cognitive Performance Between Women in the Menstrual and Pre-Ovulatory Phases

The longitudinal analysis revealed significant improvements in cognitive performance among women during the pre-ovulatory phase compared to their performance in the menstrual phase. Specifically, women demonstrated enhanced working memory, with better results in the digit span forward (T = 2.06, *p* = 0.04, r = 0.22), digit span forward max (T = 2.61, *p* = 0.01, r = 0.28), and digit span backwards max (T = 2.32, *p* = 0.02, r = 0.25) tests. Performance in attention switching also improved, as indicated by a faster completion time for the Trail Making Test B (T = 2.61, *p* = 0.01, r = 0.28). For all statistically significant results, the effect size ranged from 0.2 to 0.3, indicating a small-to-medium effect size. No significant changes were observed for other cognitive measures. Detailed results for all within-woman comparisons are presented in Table 3.

### 3.3. Differences in Cognitive Performance Between Men and Women in Two Phases of the Menstrual Cycle

The cross-sectional analysis revealed significant, phase-dependent sex differences in cognitive processing speed. An overall comparison between men (M), women in the menstrual phase (W1), and women in the pre-ovulatory phase (W2) showed significant group differences in the Trail Making Test A (H = 6.77, *p* = 0.03) and the Stroop B test (H = 6.60, *p* = 0.04).

**Figure 4:**
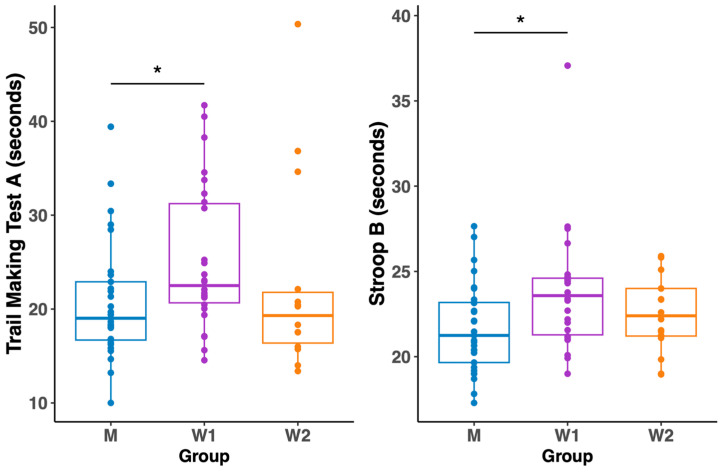
Changes in performance time in the TMT A and the Stroop test in task B between men and women in two cycle phases. *Note:* Significant differences are denoted by * *p* < 0.05; *Abbreviations:* M, men; W1, women in the menstruation phase; W2, women in the pre-ovulatory phase.

Post hoc tests specified that these differences were driven solely by the comparison between men and women in their menstrual phase (W1), where men were significantly faster on both the TMT A (*p* = 0.04) and Stroop B (*p* = 0.04). Crucially, as illustrated in Figure 4, these sex differences disappeared when women were in their high-oestradiol, pre-ovulatory phase (W2). No significant differences between the three groups were found in any other cognitive tests. Full comparative results are shown in Table 4.

### 3.4. Correlation Between Hormone Concentration and Cognitive Function

Within group W1 (the low-oestradiol phase), a relatively higher level of oestradiol was related to better performance in the Stroop interference c (cor. 0.509; *p* = 0.009). At the same time, an elevated oestradiol level was negatively correlated with the time score in the TMT A test (cor. −0.513; *p* = 0.005) in the M group.

In group W1, the higher level of progesterone was negatively related to the results in the digit span backwards test (cor. −0.407; *p* = 0.043) and Stroop interference c (cor. −0.442; *p* = 0.027). Meanwhile, in the M group, progesterone levels were positively correlated with the results in the Corsi block span forward (cor. 0.366; *p* = 0.055) and Corsi TSF (cor. 0.396; *p* = 0.037) tests.

Testosterone levels did not correlate with any cognitive test scores. No correlations were observed in the W2 group or when correlating all groups together.

## 4. Discussion

There are three main findings of the study. The first conclusion was that women’s cognitive functioning differs according to their cycle phase. We found better short-term memory capacity, working memory for auditory material, and attention during the high-oestradiol phase compared to the low-oestradiol phase in a group of the same women.

In our study, we specifically investigated the predominant effect of oestradiol on cognitive function by conducting measurements during both menstruation (low oestradiol) and the pre-ovulatory phase (high oestradiol with minimal progesterone influence). This methodological approach differs from most of the available literature, which compares menstrual and luteal phases [13,41,42,43,44,45], when both oestradiol and progesterone levels are elevated. Such methodological differences between studies create challenges in directly comparing research findings. Nevertheless, based on the available literature, we see consistency between our results and data from the literature, where young women with higher oestradiol levels showed better performance in working memory [15]. In the study by Rosenberg and Park [46], similar to our findings, performing tasks during the high-oestradiol phase was associated with improved verbal working memory, but there was no noticeable effect on spatial tasks. However, the small number of participants and the fact that the cycle phase was estimated in the Rosenberg and Park [46] study should be taken into account. Contrary to our findings, previous research (comparing women in four cycle phases) demonstrated enhanced visuospatial memory during the pre-ovulatory phase [18]. These discrepant results may reflect methodological differences in visuospatial assessment, as the cited study employed location-based tasks while our battery included sequential spatial memory (Corsi block-tapping test) and pattern recognition (visual pattern test), suggesting that cycle effects may be task-specific within the visuospatial domain.

Our findings indicating the influence of menstrual cycle phases on cognitive functions contrast with the study by Leeners et al. [27], who found no consistent associations between sex hormone levels and attention, working memory, and cognitive control in two consecutive menstrual cycles in four cycle phases. This methodologically rigorous study highlighted the problem of false positive results in this field, but several factors can explain the differences in our observations. First, we used a more extended battery of cognitive tests (in the cited study, these were the Cognitive Bias Test, Divided Attention Bimodal Task, and Corsi—in which, as in our study, they did not observe statistically significant changes). Second, our study focused exclusively on healthy young women. In contrast, approximately 34% of the sample in Leeners’ study consisted of women with endocrine disorders (endometriosis, PCOS), which may have influenced the results. Third, although authors paid particular attention to practice effects as a potential source of false results, we randomised the first assessment phase in our study, which may have controlled for this confound more effectively. It is also worth noting that a later study by Leeners and colleagues [26], using an ovarian stimulation model for infertility treatment, also found no association between very high oestradiol levels and cognitive function, suggesting that even very high levels of this hormone do not affect cognitive function directly and unambiguously. However, it should be emphasised that the model used in the 2021 study differs from the natural hormonal fluctuations in the menstrual cycle, where changes occur not only in oestradiol but also in progesterone and other hormones in a strictly defined time pattern. Nevertheless, the cited study is an important reminder of the need for cautious interpretation of results in studies on the effects of hormones on cognitive function. It highlights the value of replicating results across menstrual cycles—an aspect that should be considered in future studies.

To better understand the observed changes, as well as the discrepancies between studies focusing on cognitive tests themselves, it is worth taking a closer look at neuroimaging studies. Neuroimaging studies have demonstrated that oestradiol enhances hippocampal activation during the pre-ovulatory phase of the menstrual cycle in both verbal and spatial navigation tasks [9,47].

Oestradiol enhances glutamatergic neurotransmission and reduces GABAergic neurotransmission, creating an overall excitatory effect in the brain [6]. This increased neuronal excitability may explain oestradiol’s role in boosting neural activity during cognitive tasks in high-hormone phases of the cycle, such as the pre-ovulatory phase [7]. The enhancement of glutamatergic transmission and the reduction in GABAergic inhibition under the influence of oestradiol increases neuronal excitability, facilitating rapid and effective data processing necessary for maintaining concentration and manipulating information in working memory [6], which we observed in our study. Furthermore, oestradiol increases the dendritic spine density in the hippocampus [48,49], which may improve memory functions, including working memory. Studies also indicate that oestrogen affects the function of the dopaminergic system, which plays a key role in cognitive processes such as working memory and executive function. Higher levels of oestradiol may improve working memory performance by increasing the efficiency of information processing in the prefrontal cortex [50].

Our second key finding showed that sex differences in information processing speed and executive functioning between men and women were observed only when women were in their low-oestradiol (menstrual) phase. This phase-dependent effect was particularly evident in information processing speed (TMT A time and Stroop B time). These differences notably disappeared when women were tested during their pre-ovulatory phase, suggesting that hormonal status plays a role in modulating cognitive sex differences.

The literature presents inconsistent results regarding sex differences in attention [51,52,53,54]. The reason for this lack of clarity is that many studies investigating the cognitive differences between the sexes do not consider the hormonal changes that occur during the menstrual cycle and their impact on the results obtained. Recent neuroimaging evidence has provided insight into these hormone-dependent effects. Pletzer et al. [47] have shown that oestradiol increases hippocampal activation during the pre-ovulatory phase, which may facilitate information processing and cognitive performance. This is in line with our observation of reduced sex differences in the pre-ovulatory phase, suggesting that elevated oestradiol levels may have a compensatory function in female cognitive performance.

Furthermore, our findings complement previous research on sustained attention, where Pletzer et al. [45] observed cycle-dependent variations in attention. While their study focused on the luteal phase, showing slower response times in women compared to men during high progesterone levels, our results extend these observations by demonstrating that sex differences are particularly pronounced during the low-oestradiol phase. These findings collectively suggest that both oestradiol and progesterone play distinct roles in modulating cognitive performance, with oestradiol potentially serving a protective or enhancing function that may help eliminate baseline sex differences in cognitive processing speed.

What is concerning is the lack of differences in the visuospatial tests in our study between men and women. The literature suggests male predominance in visuospatial tasks. This advantage appears to be domain-specific, primarily documented in mental rotation [55,56] and visual motion processing tasks [53]. Our study’s absence of sex-related differences in visuospatial functions may be attributed to our test selection. Our battery included measures of visuospatial capacity and pattern retrieval (visual pattern test; VPT) and visuospatial sequential working memory (Corsi block-tapping test) rather than tasks involving mental rotation or motion processing. This methodological distinction may explain why our findings diverge from the commonly reported male advantage in specific visuospatial domains.

Our third finding has revealed complex, gender-specific associations between sex hormone levels and cognitive functions, but only during the low oestradiol phase in women. In men, oestradiol levels positively correlated with processing speed, while progesterone showed associations with enhanced spatial memory capacity. However, during the low oestradiol phase, the relationship between hormones and cognition appeared more nuanced in women. Oestradiol demonstrated a significant negative relationship with selective attention (Stroop interference), while progesterone showed an inverse pattern, correlating positively with selective attention but negatively with auditory working memory.

These seemingly contradictory findings can be understood through the underlying neurobiological mechanisms. Pletzer et al. [47] demonstrated that oestradiol and progesterone exert opposing effects on neurotransmitter systems: oestradiol enhances glutamatergic transmission while reducing GABAergic neurotransmission, whereas progesterone produces the opposite effect. This antagonistic relationship between these hormones at the neurotransmitter level may explain our observed differential effects on cognitive function. During the early follicular phase, when both hormones are at their lowest levels, the absence of oestradiol’s stimulating effect on glutamatergic transmission may contribute to decreased performance in certain cognitive domains. Conversely, progesterone’s enhancement of GABAergic inhibition could potentially impair performance through increased neural inhibition [6].

Interestingly, our study found no significant correlations between testosterone levels and cognitive performance in men or women. This finding may be explained by interpreting the results obtained in the context of the age and hormonal characteristics of our sample. The absence of testosterone effects in our study contrasts with some previous research showing testosterone’s influence on cognitive function, particularly in spatial abilities and working memory [24]. However, studies showing testosterone’s cognitive effects often focus on ageing populations [23,57,58,59]. As shown by Thilers et al. [25] in their population-based study of 35–90-year-olds, associations between endogenous testosterone levels and cognitive performance become more pronounced with age, particularly in tasks involving processing speed and spatial abilities. This moderation according to age is particularly important because testosterone levels begin to gradually decline, by about 1–2% per year, from the age of 30 [60,61], with the greatest decline observed in the sixth decade of life [24]. Our results from a young adult sample suggest that these associations may not be evident during the peak reproductive years when hormone levels are relatively stable. This is consistent with other studies investigating the relationship between testosterone levels in young adults and cognitive performance [62], including spatial abilities [63], navigation and verbal fluency tasks [64], and working memory [15]. The relationship between testosterone and cognition is complex and potentially non-linear, as demonstrated by several foundational studies [65,66], showing that both low and high testosterone levels are associated with poorer cognitive ability. In our study, the male participants showed testosterone levels within the normal age-appropriate range (laboratory norm: 8.64–29.0 nmol/l; results of participants: M = 18.95 ± 6.07 nmol/mL); the women’s testosterone variations were minimal (fluctuating within 0.45 nmol/mL between cycle phases). In people with normal testosterone levels, as in our study, the effect of this hormone on cognitive function can be challenging to observe. These findings collectively suggest that the absence of a testosterone–cognition correlation in our study may be attributed to our sample’s age range and the expected physiological hormone levels observed.

A limitation of our study is that our longitudinal assessment was restricted to two time points (menstrual and pre-ovulatory phases), which limited our ability to comprehensively assess hormonal influences throughout the menstrual cycle. To better understand the complex relationships between oestrogen and progesterone throughout the cycle, future studies should consider the three phases of the menstrual cycle. The second limitation of our study was the lack of mood assessment, which prevented us from examining how emotional state variations across the menstrual cycle might have influenced the observed cognitive differences. Recent research suggests that menstrual cycle-related cognitive changes are more pronounced in women with premenstrual dysphoric disorder (PMDD), specifically regarding executive function impairments [67,68]. Nevertheless, in our study, women with dysphoric disorder were excluded from enrolment. Future research should incorporate mood measures, as emotional states may act as moderating variables influencing the magnitude of cognitive performance differences observed between cycle phases and between sexes. A third limitation was the relatively small sample size, which may have constrained the statistical power of our findings. This was primarily due to the logistical challenges of conducting the study. Future studies with larger and more diverse samples would enhance the robustness and generalizability of the results.

## 5. Conclusions

Our findings demonstrate three key aspects of hormone–cognition interactions: (1) enhanced cognitive performance during the pre-ovulatory phase compared to the menstrual phase in women, only in verbal working memory and attention; (2) phase-dependent sex differences between men and women in processing speed are present only during women’s menstrual phase and absent during the pre-ovulatory phase; and (3) distinct hormone–cognition relationships in men and women vary according to the menstrual cycle phase. These results highlight the necessity of considering the phases of the menstrual cycle in scientific and clinical research where cognitive functions are assessed, especially in studies on sex differences.

## Figures and Tables

**Table 1:** Presentation of the information on women’s menstrual cycles.

Women (n = 42)			
Variable	M (SD)	Min Value	Max Value
Length of the menstrual cycle (days)	30.05 (2.17)	25	35
Duration of the menstrual phase (days)	5.52 (0.86)	4	7

*Abbreviations:* n, number of participants in a given group; M, mean; SD, standard deviation; Min value, minimum value in a given set; Max value, maximum value in a given set.

**Table 2:** Concentrations of hormones in groups of women and men.

Group/Phase	Testosterone (nmol/mL) M (SD)	Progesterone (ng/mL) M (SD)	Oestradiol (pg/mL) M (SD)
Women (n = 42)			
Menstrual phase	1.32 (0.45)	0.37 (0.2)	30.86 (16.74)
Pre-ovulatory phase	1.77 (0.58)	2.06 (3.92)	163.98 (115.85)
Men (n = 29)			
	18.95 (6.07)	0.35 (0.19)	24.79 (8.09)

*Abbreviations:* n, number of participants in a given group; M, mean; SD, standard deviation.

**Table 3:** Changes in the cognitive performance between women in the menstrual and pre-ovulatory phases of the cycle.

Variables	Group	N	Mean	SD	Median	Mean Rank	Sum of Ranks	T	*p*	r
Digit span forward	W_M	42	7.33	2.54	7	Negative	133.5	2.06	0.04 *	0.22
	W_PO	42	8.0	2.06	8	Positive	331.5			
Digit span forward max	W_M	42	6.24	1.38	6	Negative	93.0	2.61	0.01 *	0.28
	W_PO	42	6.74	1.27	7	Positive	313.0			
Digit span backward	W_M	42	7.0	2.35	7	Negative	167.5	1.84	0.07	0.20
	W_PO	42	7.48	2.08	7	Positive	360.5			
Digit span backward max	W_M	42	4.98	1.3	5	Negative	96.0	2.32	0.02 *	0.25
	W_PO	42	5.43	1.21	5	Positive	282.0			
TMT A time (s)	W_M	42	22.83	7.41	21.45	Negative	525.5	1.55	0.12	0.17
	W_PO	40	21.68	7.29	20.13	Positive	294.50			
TMT B time (s)	W_M	42	47.47	14.38	47.23	Negative	604.00	2.61	0.01 *	0.28
	W_PO	40	41.57	9.74	41.63	Positive	216.00			
TMT B/A time (s)	W_M	42	2.15	0.56	2.11	Negative	489.00	1.06	0.29	0.12
	W_PO	40	2.02	0.57	1.89	Positive	331.00			
Corsi block span forward	W_M	42	6.33	1.07	6	Negative	228.5	0.98	0.33	0.11
	W_PO	42	6.17	1.17	6	Positive	149.5			
Corsi TSF	W_M	42	61.9	21.01	60	Negative	409.0	0.87	0.39	0.09
	W_PO	42	58.88	21.76	54	Positive	294.0			
Corsi block span backward	W_M	42	6.60	1.29	6.00	Negative	153.00	0.27	0.79	0.03
	W_PO	42	6.57	0.80	6.00	Positive	172.00			
Corsi TSB	W_M	42	62.10	17.23	60.00	Negative	204.50	1.81	0.07	0.20
	W_PO	42	67.05	17.71	60.00	Positive	425.50			
VPT max	W_M	41	10.32	1.86	10	Negative	172.0	1.52	0.13	0.17
	W_PO	42	10.76	1.86	11	Positive	324.0			
VPT mean	W_M	41	9.76	1.68	10.30	Negative	202.50	1.85	0.07	0.20
	W_PO	42	10.18	1.76	10.00	Positive	427.50			
VMT Vis Mem	W_M	41	16.22	1.26	16.00	Negative	221.50	0.53	0.60	0.06
	W_PO	40	16.38	1.41	17.00	Positive	274.50			
VMT Seq Mem	W_M	41	14.61	1.52	15.00	Negative	163.50	1.91	0.06	0.21
	W_PO	40	15.28	1.63	15.00	Positive	364.50			
Stroop A time (s)	W_M	42	28.28	4.67	27.92	Negative	517.5	0.83	0.41	0.09
	W_PO	42	27.85	4.1	26.45	Positive	385.5			
Stroop B time (s)	W_M	42	22.89	3.50	22.16	Negative	477.50	0.33	0.75	0.04
	W_PO	42	22.48	2.42	22.13	Positive	425.50			
Stroop C time (s)	W_M	42	44.45	9.89	44.75	Negative	606.00	1.93	0.05	0.21
	W_PO	42	42.21	8.03	41.53	Positive	297.00			
Stroop D time (s)	W_M	42	49.10	9.61	48.65	Negative	540.00	1.11	0.27	0.12
	W_PO	42	47.69	9.61	47.74	Positive	363.00			
Stroop interference	W_M	42	21.56	7.8	21.44	Negative	598.0	1.83	0.07	0.20
	W_PO	42	19.72	7.69	19.79	Positive	305.00			
Stroop interference a	W_M	42	16.17	7.50	13.62	Negative	596.00	1.81	0.07	0.20
	W_PO	42	14.35	6.08	13.70	Positive	307.00			
Stroop interference b	W_M	42	−2.06	8.37	−2.65	Negative	552.50	1.26	0.21	0.14
	W_PO	42	−2.65	7.84	−3.04	Positive	350.50			
Stroop interference c	W_M	42	4.65	7.94	4.65	Negative	429.00	0.28	0.78	0.03
	W_PO	42	5.48	8.44	5.21	Positive	474.00			

*Note:* Values marked with an asterisk (*) indicate the level of statistical significance (*p* < 0.05). *Abbreviations:* W_M, women in the menstruation phase; W_PO, women in the pre-ovulatory phase; N, the number of participants in a given group.; SD, standard deviation; T, the test statistic for the Wilcoxon signed-rank test; *p*, the *p*-value, representing the probability of obtaining the observed results under the null hypothesis; *p* < 0.05, indicates statistical significance, meaning there is less than a 5% probability that the observed effect occurred by chance; r, the effect size; DSF, digit span forward; DSB, digit span backward; TMT A, Trail Making Test A; TMT B, Trail Making Test B; TMT B/A, Trail Making Test B/A Ratio; CORSI TSF, Corsi Total Score Forward; CORSI TSB, Corsi Total Score Backward; VPT Max, Visuospatial Test Maximum, the highest level of performance achieved in a visuospatial test; VPT Mean, Visuospatial Test Mean, the average score in a visuospatial test, reflects overall performance; VMT Vis Mem, Visual Memory Task—Visual Memory; VMT Seq Mem, Visual Memory Task—Sequential Memory. Stroop interference, the difference between the times taken to complete Stroop tests C and B (Stroop C − Stroop B); Stroop interference a, the difference between the times taken to complete Stroop tests C and A (Stroop C − Stroop A); Stroop interference b, difference between the times taken to complete Stroop test D and the sum of the times taken to complete Stroop tests A and B (Stroop D − (Stroop A + Stroop B); Stroop interference c, difference between the times taken to complete Stroop tests D and C (Stroop D − Stroop C).

**Table 4:** Differences in cognitive performance between men and women in the two phases of the menstrual cycle.

**Cognitive Test**	**Group**	**Statistics**					
		**N**	**Mean Rank**	**Median**	**IQR**	**KW Statistic**	***p***
Digit span forward							
	W1	26	30.58	7	2	2.96	0.23
	W2	16	40.22	8	4		
	M	29	38.53	8	4		
Digit span forward max							
	W1	26	30.88	6	2	2.67	0.26
	W2	16	39.47	6.5	2		
	M	29	38.67	7	2		
Digit span backward							
	W1	26	31.27	7	3	2.21	0.33
	W2	16	38.25	8	3		
	M	29	39.00	8	4		
Digit span backward max							
	W1	26	30.96	5	2	2.99	0.22
	W2	16	36.38	5.5	2		
	M	29	40.31	6	3		
TMT A time (s)							
	W1	26	43.08	22.5	11.26	6.77	0.03 *
	W2	14	30.50	19.31	9.32		
	M	29	29.93	19.02	6.71		
TMT B time (s)							
	W1	26	37.27	47.75	16.76	0.54	0.76
	W2	14	34.07	47.91	15.19		
	M	29	33.41	44.55	17.78		
TMT B/A time (s)							
	W1	26	30.88	1.89	0.56	1.97	0.37
	W2	14	35.43	2.16	1.12		
	M	29	38.48	2.06	1.21		
Corsi block span forward							
	W1	26	37.12	6	2	0.15	0.93
	W2	16	35.97	6	3		
	M	29	35.02	6	2		
Corsi TSF							
	W1	26	36.58	54	32	0.22	0.90
	W2	16	37.44	57	50		
	M	29	34.69	54	30		
Corsi block span backward							
	W1	26	32.79	6	1	2.27	0.32
	W2	16	42.00	7	2		
	M	29	35.57	6	1		
Corsi TSB							
	W1	26	29.87	57	12	4.37	0.11
	W2	16	42.88	66.5	31		
	M	29	37.71	60	23		
VPT max							
	W1	26	34.62	11	3	1.03	0.60
	W2	16	40.53	11	2		
	M	29	34.74	10	3		
VPT mean							
	W1	26	34.60	10.3	3.07	0.56	0.76
	W2	16	39.31	10.15	2.6		
	M	29	35.43	9.67	2.16		
VMT Vis Mem							
	W1	26	33.87	16	2	0.80	0.67
	W2	14	32.25	16	2		
	M	29	37.34	16	1		
VMT Seq Mem							
	W1	26	33.81	14.5	3	0.40	0.82
	W2	14	33.57	14.5	3		
	M	29	36.76	15.00	2		
Stroop A time (s)							
	W1	26	39.69	28.88	6.8	1.35	0.51
	W2	16	33.06	28.18	7.51		
	M	29	34.31	28.44	4.86		
Stroop B time (s)							
	W1	26	43.37	23.58	3.47	6.60	0.04 *
	W2	14	36.59	22.4	2.86		
	M	29	29.07	21.25	3.75		
Stroop C time (s)							
	W1	26	37.96	45.35	13.23	0.38	0.83
	W2	16	34.38	46.27	15.17		
	M	29	35.14	44.00	9.85		
Stroop D time (s)							
	W1	26	36.38	52.70	13.76	0.35	0.84
	W2	16	33.38	49.28	13.6		
	M	29	37.10	52.09	9.84		
Stroop interference							
	W1	26	35.92	22.37	13.95	0.45	0.80
	W2	16	33.28	23.09	14.45		
	M	29	37.57	23.68	9.99		
Stroop interference a							
	W1	26	36.50	14.11	12.8	0.17	0.92
	W2	16	34.13	15.54	10.59		
	M	29	36.59	15.61	8.71		
Stroop interference b							
	W1	26	32.38	−2.82	15.18	2.44	0.30
	W2	16	33.59	0.13	12.51		
	M	29	40.57	1.34	9.19		
Stroop interference c							
	W1	26	32.08	4.30	12.68	1.49	0.47
	W2	16	37.81	6.96	9.55		
	M	29	38.52	7.28	9.59		

*Note:* Values marked with an asterisk (*) indicate the level of statistical significance (*p* < 0.05). *Abbreviations:* W_1, women in the menstruation phase; W_2, women in the pre-ovulatory phase; M, men; N, the number of participants in a given group; IQR, Interquartile Range—a measure of statistical dispersion, representing the range between the first and third quartiles (Q1–Q3); KW Statistic, Kruskal–Wallis Test Statistic; *p*, the *p*-value, representing the probability of obtaining the observed results under the null hypothesis; *p* < 0.05, indicates statistical significance, meaning there is less than a 5% probability that the observed effect occurred by chance; DSF, digit span forward; DSB, digit span backward; TMT A, Trail Making Test A; TMT B, Trail Making Test B; TMT B/A, Trail Making Test B/A Ratio; CORSI TSF, Corsi Total Score Forward; CORSI TSB, Corsi Total Score Backward; VPT Max, Visuospatial Test Maximum, the highest level of performance achieved in a visuospatial test; VPT Mean, Visuospatial Test Mean, the average score in a visuospatial test, reflecting overall performance; VMT Vis Mem, Visual Memory Task—Visual Memory; VMT Seq Mem, Visual Memory Task—Sequential Memory. Stroop interference, the difference between the times taken to complete Stroop tests C and B (Stroop C − Stroop B); Stroop interference a, the difference between the times taken to complete Stroop tests C and A (Stroop C − Stroop A); Stroop interference b, difference between the times taken to complete Stroop test D and the sum of the times taken to complete Stroop tests A and B (Stroop D − (Stroop A + Stroop B); Stroop interference c, difference between the times taken to complete Stroop tests D and C (Stroop D − Stroop C).

## Data Availability

The data supporting the conclusions of this article are included within the article and its Supplementary Materials.

## Supplementary Materials

The following supporting information can be downloaded at: https://www.mdpi.com/article/10.3390/biology14081060/s1. The Supplementary Materials contain raw data on hormone levels and cognitive test results.
